# Brain Correlates of Non-Symbolic Numerosity Estimation in Low and High Mathematical Ability Children

**DOI:** 10.1371/journal.pone.0004587

**Published:** 2009-02-24

**Authors:** Yulia Kovas, Vincent Giampietro, Essi Viding, Virginia Ng, Michael Brammer, Gareth J. Barker, Francesca G. E. Happé, Robert Plomin

**Affiliations:** 1 Institute of Psychiatry, King's College London, London, United Kingdom; 2 Division of Psychology and Language Sciences, University College London, London, United Kingdom; 3 Department of Psychology, Goldsmiths, University of London, London, United Kingdom; University of Minnesota, United States of America

## Abstract

Previous studies have implicated several brain areas as subserving numerical approximation. Most studies have examined brain correlates of adult numerical approximation and have not considered individual differences in mathematical ability. The present study examined non-symbolic numerical approximation in two groups of 10-year-olds: Children with low and high mathematical ability. The aims of this study were to investigate the brain mechanisms associated with approximate numerosity in children and to assess whether individual differences in mathematical ability are associated with differential brain correlates during the approximation task. The results suggest that, similarly to adults, multiple and distributed brain areas are involved in approximation in children. Despite equal behavioral performance, there were differences in the brain activation patterns between low and high mathematical ability groups during the approximation task. This suggests that individual differences in mathematical ability are reflected in differential brain response during approximation.

## Introduction

Several brain regions show increased brain activation during numerical approximation tasks when compared to control tasks. These include intraparietal sulcus, inferior and superior frontal gyri, as well as other co-ordinates within the precentral, dorsolateral and superior prefrontal regions[1]–[2]. These regions also show increased activation with increased complexity of the approximation performed in the scanner [1]. According to one influential hypothesis, these regions, and in particular the horizontal segment of the intraparietal sulcus (hIPS), are the loci of a dedicated, domain-specific number system, subserving operations with both symbolic and nonsymbolic stimuli [3]. Several studies, using different fMRI paradigms to compare activation between numerical activity and control tasks have replicated the involvement of hIPS in both symbolic and non-symbolic numerical judgments [4]. For example, a recent study found increased activation in the hIPS and in frontal regions, irrespective of whether digits, dots, or number words were used in numerical judgments [4]. The authors concluded that these results support the idea that symbols acquire meaning by linking neural populations coding symbol shapes to those holding nonsymbolic representations of quantities. They also suggested that it is likely that symbolic and concrete depictions of number are linked together in the adult human brain in the form of notation-independent assemblies of neurons coding for number at a purely conceptual level (cardinality).

However, a recent study [5], using nonsymbolic stimuli (dots), did not find higher parietal activations for number than for non-number comparison tasks. This study specifically tested whether the same parietal areas were involved in the numerosity judgments involving dot arrays and those involving Arabic numerals. The results of the study posed a serious challenge to the hypothesized single amodal numerosity representation, in that different regions were activated during tasks involving dots and numerals. For example, no significant BOLD fMRI activations in hypothesized numerosity areas were found for the dots numerosity judgment above those seen in the difficulty-matched color control task. Indeed, many of the hypothesized numerosity areas showed significantly more activation during the color control task. Moreover, the two activations for the non-symbolic numerosity judgment task were not in the hypothesized numerosity areas, but in inferior temporal gyrus and in the middle occipital gyrus. Neither of these activations was close to any area previously implicated in number cognition. In addition, even within one study the areas that were more activated during color or numerical comparisons did not replicate across very similar experiments. In light of these inconsistent findings, more numerosity studies looking at the whole brain rather than focusing on the regions of interest are required.

Although the body of developmental research into neural correlates of numerosity is growing [6]–[9], most fMRI work on numerosity judgments has focused on adults. Behavioral studies have shown that children are sensitive to the numerical attributes of stimuli from a very young age [10]. For example, in both children and adults the capacity for approximate non-symbolic numerical estimation shows the same signature of ratio-dependent discrimination [7]. The first fMRI study investigating brain activity related to numerosity judgments in children and adults found evidence for the involvement of hIPS in the non-symbolic numerical activity in both age groups [7]. In an event-related passive viewing paradigm, the hIPS showed increased activation (above both rest and above the control task) during non-symbolic numerical activity in both adults and 4-year-old children. In adults, the number-related activity extended beyond hIPS into the inferior and superior parietal lobules. In children, the activation was found in and around the right hIPS and right superior parietal lobule. The activations were also found in the left precentral gyrus, left superior frontal gyrus, left medial frontal gyrus, left inferior parietal lobule, and right middle frontal gyrus. The authors stated that they had no a priori hypotheses regarding the roles of these latter regions in numerical processing and did not offer any further discussion of these findings, including the finding of a more distributed network of activations in children.

Another recent study compared the neural correlates of nonsymbolic magnitude judgments between healthy children and adults using fMRI [6]. In children, the difficulty of the task (smaller distance) was associated with the increased activation in the right DLPFC, left inferior frontal gyrus, and left IPS. In adults, the left and right IPS, right superior frontal gyrus, left and right anterior cingulate gyrus, posterior cingulate gyrus, and LIFG were involved. The test of group differences resulted in greater effects of numerical distance on the left intraparietal sulcus in adults than in children, suggesting that this area may undergo significant age-related changes.

Limited research to date has addressed the issue of individual variation in relation to mathematical ability and associated brain activity. fMRI studies with patients with dyscalculia have shown decreased or abnormally modulated activity or decrease in the grey matter density in parietal cortices in people with dyscalculia (reviewed in [8]). One recent study comparing children with developmental dyscalculia (DD) and typically achieving schoolchildren found that during a non-symbolic approximation task (magnitude comparison) children with DD activated a network of primary and secondary visual areas including middle occipital gyrus, fusiform gyrus (FG), lingual gyrus, and cuneus [8]. In the right hemisphere, the network extended into the parietal lobe along with the intraparietal sulcus. Control children in this study showed the same pattern of activation as the children with DD, but also showed additional bilateral parietal activation foci in the hIPS. When the results were corrected for multiple comparisons, no differences in activations between children with DD and typically achieving children were found on this task. However, group differences surviving multiple comparisons were observed with another approximation task used in the study (approximate calculation with numerals). The authors did not offer hypotheses regarding why such differences were observed with one but not the other approximation task.

Another recent study [9] compared the neural correlates of basic numerical processing (non-symbolic numerosity judgment) in children with DD and in typically developing children using fMRI. The results showed a stronger distance effect in the IPS and in the fusiform gyrus in the control group than in DD group. The dyscalculic group also showed a greater deactivation for small distances. The authors concluded that specific abnormalities existed in the functional neuroanatomy underlying numerical magnitude processing in developmental dyscalculia. Clearly, the two studies examining potential differences in neural correlates of numerical processing between typically developing children and children with developmental dyscalculia provided inconsistent results.

The present study is designed to address these inconsistencies. The study used a whole-brain approach in order to explore the distributed network of brain areas involved in non-symbolic approximate numerical judgment. Two groups of 10-year-old children selected to have stable low or high mathematical ability (as assessed on three occasions at seven, nine, and ten years of age) were studied. This is thus the first study to date to examine individual differences in brain processing of non-symbolic approximation using longitudinal data to select two stable extreme (rather than poor vs. average) groups of children of the same age. This method of sample selection should maximize putative neural differences between groups. The main aims of this study were: (1) to investigate the brain mechanisms underlying non-symbolic approximate numerosity judgments in 10-year-old children; (2) to assess whether individual differences in mathematical ability are associated with differential brain correlates during approximation in a sample selected on their stable mathematical performance across a 3-year span.

## Results

### Behavioral Results

For each task administered in the scanner, descriptive statistics for accuracy and reaction times (RT) to correct responses were obtained for low (N = 13) and high (N = 13) ability groups. Five children from the low ability group were excluded from the subsequent analyses because they did not reach the criteria of at least 60% accuracy on the easiest condition (1∶2 Dot Ratio). Following exclusions, the low and high mathematical ability groups were compared on all Dot Ratio, Dot Setsize, and Color Control conditions and no group differences emerged in either accuracy or RT. The only exception was the Medium Setsize condition, in which low ability group was significantly lower in accuracy than the high ability group.

Tables 1 and 2 present descriptive statistics for percentage of correct responses and RT to correct responses for the nine conditions and for the two mathematical ability groups. A series of mixed design ANOVAs and pairwise comparisons were performed on the data from the low and high mathematical ability groups. All reported significant results were adjusted for multiple comparisons (Bonferroni correction). ANCOVA analyses were also performed with IQ as a covariate, as the low and high groups differed on cognitive ability. For all analyses results of ANCOVA showed that covarying for IQ did not change the pattern of results.

**Table 1:** Behavioral Results: % correct responses averaged across 3 runs for low and high mathematical ability groups.

Task	Group	Minimum	Maximum	Mean	Std. Deviation
Dot Ratio 1∶2	High	58.30	88.90	75.32	9.49
	Low	65.00	88.90	75.48	8.39
Dot Ratio 2∶3	High	30.60	80.60	63.95	16.43
	Low	55.60	80.60	68.38	8.54
Dot Ratio 3∶4	High	36.10	80.60	61.96	12.14
	Low	41.70	77.80	60.85	12.20
Dot Ratio 4∶5	High	47.20	75.00	56.20	7.62
	Low	40.00	77.80	54.66	14.30
Dot Ratio 7∶8	High	38.90	80.60	54.70	10.24
	Low	44.40	61.10	52.26	6.58
Dot Setsize Small	High	40.00	80.00	67.05	10.34
	Low	63.80	86.70	73.05	8.33
Dot Setsize Medium	High	52.50	83.80	70.02	9.91
	Low	51.30	76.30	64.10	8.20
Dot Setsize Large	High	55.00	83.80	67.62	9.04
	Low	58.80	78.80	70.94	7.48
Color Task	High	58.30	96.70	81.95	12.21
	Low	67.70	95.00	87.00	8.50

*Note.* N (low mathematical ability group) = 8; N (high mathematical ability group) = 13.

**Table 2:** Behavioral Results: RTs to correct responses averaged across 3 runs for low and high mathematical ability groups.

Task	Group	Minimum	Maximum	Mean	Std. Deviation
Dot Ratio 1∶2	High	228.00	735.00	469.77	136.37
	Low	160.00	625.00	458.88	144.79
Dot Ratio 2∶3	High	253.00	717.00	497.46	127.37
	Low	218.00	601.00	455.25	128.22
Dot Ratio 3∶4*	High	305.00	723.00	520.77	142.36
	Low	139.00	660.00	462.88	179.32
Dot Ratio 4∶5	High	283.00	744.00	535.38	128.34
	Low	197.00	661.00	495.00	160.68
Dot Ratio 7∶8	High	281.00	813.00	537.85	143.64
	Low	201.00	651.00	498.63	141.61
Dot Setsize Small	High	259.00	736.00	498.92	125.29
	Low	229.00	652.00	444.00	139.12
Dot Setsize Medium	High	256.00	765.00	515.00	135.28
	Low	272.00	610.00	451.62	116.34
Dot Setsize Large	High	287.00	727.00	492.15	131.03
	Low	214.00	634.00	461.63	134.14
Color Task	High	274.00	746.00	486.54	138.92
	Low	347.00	634.00	489.13	102.64

*Note.* N (low mathematical ability group) = 8; N (high mathematical ability group) = 13.

* Significant difference between high and low groups.

A 2 (group: low vs. high mathematical ability)×5 (Ratio: 1∶2, 2∶3, 3∶4, 4∶5, and 7∶8) mixed model ANOVA was performed on accuracy scores (percentage of correct responses). There was no main effect of Group on accuracy (F = .001, df = 1, p = .978), but a significant main effect of Ratio was observed (F = 19.58, df = 4, p = .000). A polynomial contrast was performed on these data, and showed that the main effect of Ratio was linear (F = 113.32, df = 1, p = .000), in that the accuracy decreased with decreased numerical distance between the two sets of dots. Simple pairwise comparisons revealed that the 1∶2 ratio was significantly easier than all other ratios (p<.005). A significant difference was also observed between the 2∶3 ratio and the 7∶8 ratio (p = .001). The rest of the pairwise comparisons were not significant (p<.05), suggesting that a clear ratio effect was only present in comparing ratios with large numerical distance between the two sets of dots (1∶2 and 2∶3) to other ratios, and not smaller ratios to each other, No significant interaction between Ratio and Group (F = .457, df = 1, 4 p = .767) was found.

The second ANOVA compared the same conditions as the first one, with the dependent variable in this case RT to correct responses. Results showed a significant main effect of Ratio (F = 5.03, df = 4, p = .001). A polynomial contrast was performed on these data, and showed that the main effect of Ratio was linear (F = 26.46, df = 1, p = .000), in that the RTs increased with decreased numerical distance between the two sets of dots. Simple pairwise comparisons revealed that 1∶2 ratio problems took significantly less time to solve than the 3∶4 (p = .015) and 7∶8 ratios (p = .010). The rest of the pairwise comparisons were not significant (p<.015), again suggesting that a clear ratio (or numerical distance) effect was only present in comparing a ratio with a very large numerical distance to much smaller ratios, and not smaller ratios to each other. There was no effect of Group on RT (F = .391, df = 1, p = .539), and no significant interaction between Ratio and Group (F = .652, df = 4, p = .627).

The third ANOVA was 2 (group: low vs. high mathematical ability)×3 (Setsize: Small, Medium, and Large) on accuracy (percentage of correct responses). Results showed no significant main effects of Group (F = .118, df = 2, p = .735) or Setsize (F = .105, df = 2, p = .749). However a Group×Setsize interaction was significant (F = 5.02, df = 2, p = .018). Examining profile plots for this interaction revealed that for high ability group the accuracy was the same in all Setsize conditions. However, for the low mathematical ability group the Medium Setsize condition was more difficult than either Small or Large Setsize. As we have no hypotheses regarding these differences, no post-hoc pairwise comparisons were performed on these data.

The fourth ANOVA compared the same conditions as the third one, with RT to correct responses as the dependent variable. No significant main effects or interactions emerged: Group (F = .740, df = 1, p = .401); Setsize (F = .859, df = 2, p = .431); Setsize by Group Interaction (F = 1.776, df = 2, p = .183).

Two univariate ANOVAs were also run on accuracy and ‘RT to correct responses’ data in the Control Task condition (Color Matching task), with two levels of the between-group variable (Group). There were no significant main effects of Group in either accuracy (F = .198, df = 1, p = .660) or RT (F = 1.112, df = 1, p = .302).

### Conclusions from behavioral data analyses

The results replicated previous findings in adults [11] in that 10-year-old children's performance (accuracy and RT) was influenced by the distance between numerosities of the compared arrays (Ratio effect), but not the absolute Setsize of the compared displays. This was true for both low and high ability groups. The only exception to this was the finding that low ability children seemed to make more mistakes in the medium size displays than in either small or large setsize displays on which they were also faster than the high ability group. The effect of Ratio was linear, in that accuracy decreased and RT increased with decreased ratio difference. Pairwise comparisons revealed that the largest ratio (1∶2) was by far the easiest condition, and that it was significantly easier than all other ratios. The only other significant ratio effect also involved a large ratio difference (from 2∶3 to7∶8). These results suggest that Distance (Ratio) effect becomes smaller as numerical differences become smaller.

As described above, we had to exclude five participants from the low achievement group due to evidence of chance performance on the easiest task. However, when all participants were included, pairwise comparisons between low and high ability groups on accuracy and RT on all levels of the ratio resulted in non-significant average differences (results available from the authors). Similarly, for the children included in the final analyses, the two mathematical ability groups did not differ in accuracy or RT on the non-symbolic numerosity judgment task employed in this study. These results suggest that scholastic mathematical achievement (used as selection criteria) is only weakly associated with speed and accuracy of non-symbolic numerosity judgments in 10-year-old children.

### Imaging Results

#### Combined activations across all participants

Multiple areas showed increased activation during the approximate numerosity judgment task (relative to the color control task), including primary visual (parastriate) cortex/fusiform gyrus (BA 19), IFG, middle temporal gyrus (BA19), middle temporal gyrus (BA37), corpus callosum or cingulate gyrus (BA29), frontal pole (BA 10), junction of MTG/ITG (BA 37/19), post cingulate gyrus (BA 23), anterior cingulate gyrus BA 32), superior temporal gyrus/insula (BA 22), and dorsomedial frontal cortex (BA9). Some areas showed decreased activation in comparison to the color control task, including primary visual (peristriate) cortex (BA 18), primary visual (striate) cortex/cerebellum (BA 17), primary visual (peristriate) cortex (V1, V3)/lingual gyrus (BA 18), cerebellum (BA 71), middle temporal gyrus (BA 21), and middle/inferior temporal gyrus (BA 20/21).

#### Interactions

**Figure 1:**
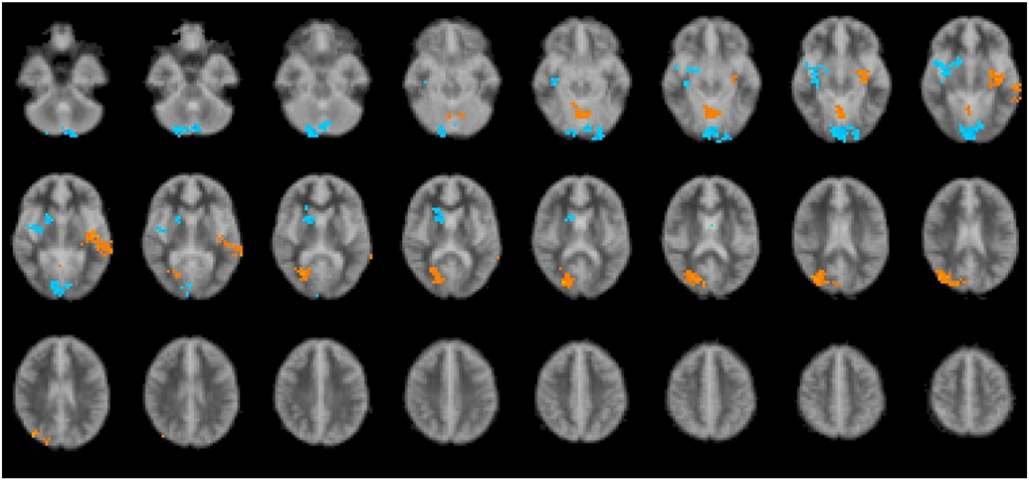
Combined brain map (Radiological format). Brain areas in all conditions in which Low mathematical ability group showed higher activation than the High mathematical ability group are in blue. Brain areas in all conditions in which High mathematics ability group showed higher activation than the Low mathematical ability group are in yellow.

The principal question we were interested in examining was the following. Is the variation in fMRI response as a function of task difficulty dependent on whether the children have high or low mathematical ability? In order to answer this question we carried out the following analysis. For each subject, at each voxel, a regression slope was calculated between difficulty level and the corresponding fMRI responses. We then entered these regression slopes to a subsequent analysis of variance to test the existence of a significant main effect of group (high vs. low mathematical ability) on the regression slopes. Figure 1 shows the results of this analysis. The regions for which the activation was significantly (correcting for multiple testing) stronger for the high ability group are shown in red. The three regions were: cerebellum (0, −63, −10), left claustrum (−33, −15, −33), and right calcarine sulcus (25, −67, 7). The regions for which the activation was stronger for the low ability group are shown in blue. The two regions were: left lingual gyrus (−4, −81, −7), and right thalamus or possibly white matter (18, 11, 10).

In addition, the average BOLD response values for each cluster were extracted for 18 ROIs selected from the previous literature as coordinates indexing the numerosity-associated activity in the hIPS. None of the ROIs showed increased or decreased activation in our study (the plotted results are available from the authors).

## Discussion

The first aim of this study was to investigate the brain correlates of non-symbolic approximation in 10-year-old-children. Numerosity related brain activation was widely distributed in the brain and included cerebellum, insula, superior temporal gyrus, hippocampus, medial frontal lobe, dorsolateral prefrontal cortex, cingulate gyrus, and superior longitudinal fasciculus. Many of these brain areas have been implicated in non-symbolic and symbolic numerical judgments in previous studies [5], [7]. However, due to a tendency in the literature towards focusing on parietal regions of interest, and in particular the hIPS, there is currently no comprehensive hypothesis about the involvement of the areas outside hIPS, as reported in this study and by others before us.

The results of the present study suggest that approximate non-symbolic judgment is subserved by a widely distributed brain network. Some parts of this network appear to support numerical judgments in general (as shown by activation in both low and high ability group), whereas others may subserve individual differences in numerical ability, as manifested by magnitude differences in brain activation between low and high mathematical ability children.

Similar to [5], we found no significant increased or decreased activation related to non-symbolic numerical estimation in inferior parietal areas of the brain. Our finding goes against the hypothesis that hIPS is the main brain area subserving approximation. One potential explanation for this finding is that the regions of parietal cortex (hIPS) that were previously suggested to play a crucial role in numerosity coding were active also during the color (control) task. Participants might have ignored instructions to focus on color rather than numerosity, or neural populations in the hIPS could be automatically activated by the passive viewing of the dot arrays in the color task. The task employed in this study does not allow us to test this hypothesis. However, many areas did show more activity during the dot task, suggesting that these areas could be reasonable candidates for areas important in numerosity processing.

As most previous research involved adults, the failure to find hIPS involvement in our study could be argued to reflect a developmental pattern of the emergence of specialization in the brain. However, in the light of the previous findings of the involvement of the hIPS in a similar task in children [6], [7] this explanation faces some difficulty. If numerosity judgments are associated with subtle changes in brain activity over a widely distributed network of brain areas, then the inconsistencies in the literature could be explained by relative lack of power afforded by small scale neuroimaging studies to replicate specific findings. The selective focus on specific regions of interest, at the expense of reporting data from the whole brain, offers an additional and related explanation to the inconsistent findings (see [12] for discussion).

A novel aspect of our study was the investigation of whether differences in stable scholastic mathematical ability were associated with differences in approximation-related brain activity. We found group differences between low and high ability children in brain activity in several areas, including those implicated by previous research, but not in the hIPS. The differences were observed in both directions, so that low ability group showed over-activation in some areas and under-activation in other areas. These results contrast with those of [8], who did not find any differences in activation between children with DD and controls on a non-symbolic magnitude judgement task. Our findings also contrast with [9] who found significant group differences between typically developing and dyscalculic children in the IPS and FG regions. Clearly, more research with larger samples is needed to establish which findings are replicable.

In the present study, one area that showed more activity in the high ability children was cerebellum. This area has been previously implicated in several studies of numerosity [13]–[14]. Another area that showed stronger activation for the high ability group was the left claustrum. This area was previously implicated in tasks such as congruency of number words (bilaterally) and distance effect (left claustrum) [15]. This area is particularly interesting since it is thought to contain inputs from and projections to all regions of the cortex [16]. Finally, the right calcarine sulcus showed stronger activation for the high ability group. Close left and right occipital areas (e.g., lingual gyrus and cuneus) have been previously implicated in approximation and mental rotation tasks [13], [17].

Left lingual gyrus showed increased activation in our low ability group. Previous studies suggest that right occipital/cuneus areas very close to the lingual gyrus are involved in magnitude comparison and in calculation tasks in participants without mathematical problems [1], [13]. Finally, the right thalamic/white matter area showed stronger activation in the low ability children in our study. A similar thalamic area on the left has been previously implicated in calculation [18].

Previous and current findings suggest that some degree of laterality might exist in the networks associated with numerosity in both healthy adults, and in high vs. low mathematical ability children. Our results suggest that high ability children show higher activation in the LEFT claustrum area (−33, −15, −3), whereas low ability children show higher activation in the nearby RIGHT area (18, 11, 10). However, these regions may not represent the exact same anatomical locations, despite their apparent laterality, and so not only the laterality, but also the specific regions seem to differ between the groups. One previous study found an association between calculation and the activity in the left thalamus (−12, −14, 8) in healthy adults, which is on the opposite side from our low-ability children activation.

The high ability children also showed increased activation in the RIGHT calcarine sulcus (25, −67, 7), whereas the low ability children showed higher activation in the LEFT lingual gyrus (−4, −81, −7). Again, the exact anatomical locations were not the same for the two groups, preventing us from further speculation regarding possible laterality. However, previous research implicated similar areas. For example, [1] found the LEFT thalamus to be associated with approximate judgment in healthy adults (−20, −8, 16). In our study, a nearby but RIGHT hemisphere area (18, 11, 10) was significantly stronger activated in the low ability children. In addition, the same study found that a right cuneus (4, −76, 8) showed association with calculation (above a matching control task) in healthy adults. In our study a similar area on the LEFT (−4, −81, −7) showed an association with approximation in the low ability children. These results suggest that lateralization may play a role in the distinction between low and high mathematical ability children in terms of their brain activation.

Another recent study [15] showed an association between number word task and both right (31, 11, 4) and left (−31, 0, 4; −31, 13, 6) claustrum in healthy adults. In our study we found the association between approximation and this area on the LEFT in high ability children. Yet another study, [17], showed the involvement of the LEFT occipital area (−22, −86, −14) in the dot enumeration task in healthy adults. In our study, a similar area was active in the high ability children, but on the RIGHT (25, −67, 7). Cerebellum is another area that has been previously implicated in many studies. For example, [13] found activation associated with approximation in the RIGHT cerebellum in healthy adults (0, 68, −22). Another study [14] with healthy adults found similar cerebellum/lingual gyrus association in the LEFT cerebellum (0, −84, −20). We found a similar area activated in our high ability children (0, −63, −10). To summarise, previous results have not been consistent in terms of laterality or specific areas implicated. Moreover, some of the observed differences might be partially explained by a developmental change. More research is needed in order to investigate these effects further.

The results of this study allowed us to distinguish between two hypotheses: (1) if a network of neural activity emerges for approximate judgment vs. control condition, but no group differences in these areas are observed, this would support the idea that there is a neural network that supports non-symbolic approximation judgments irrespective of overall mathematical ability, while individual differences in mathematical ability are reflected in a different neural network (not assessed in our study); (2) if neural activity differences between the low and high mathematical ability groups are found in the absence of behavioral differences on a specific task, this suggests that mathematical ability status is reflected in the activation differences in at least some areas supporting non-symbolic approximate judgment. Although our results support the second hypothesis, the direction of the association remains unknown. Either small differences across a wide brain network lead to the individual differences in mathematical performance or differences in mathematical performance (caused by multiple genetic and environmental factors) cause the observed differences in activation during approximate judgment task.

The results of this study also suggest that different neural mechanisms may be involved in approximation per se and in individual differences in mathematical ability. This is suggested by the non-overlapping brain areas active in approximation vs. baseline and low vs. high ability comparisons. This finding could reflect a dissociation (or partial dissociation) between the areas subserving invariable (species universal) ability to use approximate judgment, and the areas subserving mechanisms by which variation in this ability arises among individuals.

It has recently been suggested that learning disabilities reflect the extremes of the same brain and cognitive processes responsible for normal variation [12], [19]. Recent genetic research has suggested that cognitive abilities and disabilities are influenced by many genes of small effect. This might mean that to the extent that normal variation in abilities are driven by genetic factors, many neural processes of small effect mediate the effects of genes on cognition [12]. Much more research in this area is needed that involves large samples in order to gain enough statistical power to detect processes of small effects in multiple brain areas at the whole brain level of analysis, to identify and replicate the complex neural networks suggested (but not established) by the existing literature.

## Materials and Methods

### Ethics Statement

An approval from the King's College Research Ethics Committee has been obtained to conduct research using TEDS sample. A written informed consent for the study was obtained from each family prior to testing.

### Participants

Participants were 10-year-old children (17 boys; 9 girls), part of the Twins Early Development Study (TEDS) (see [20] for a detailed description of TEDS). All participants had normal or corrected-to-normal vision and were screened against neurological, medical, and psychiatric diseases. As the TEDS sample is population based, participants for the low and high mathematical ability groups were selected based on a quantitative cut-off based on two very different measures of mathematics at age 7 and age 10. For the low ability group, the initial pool of participants was composed of all individuals who scored 1.5 SD or more below the population mean on a mathematics score at 7 years of age (composite of year-long teacher ratings of three different aspects of mathematics) and who also scored more than 1.5 SD below the population mean on a composite mathematics score at 10 years of age (web-based individual assessment). High mathematical ability was defined as at least 1SD above the population mean on the same two measures at the same two ages. The groups were thus selected to have either a stable low mathematical ability or a stable high mathematical ability across 3 years. From this pool, children with low IQ (more than 1SD below the population mean) and high Hyperactivity scores (above 6) were excluded (see Appendix S1.1 for a description of all measures; for detailed description of all measures used for selection in this study see [20]. The final sample included 14 low mathematical ability and 14 high mathematical ability children (see Appendix S1.2 for details of selection and recruitment). Of these, 13 low ability and 13 high ability children successfully completed the scanning sessions. The low ability group included nine boys and four girls; the high ability group included eight boys and five girls. Four of the children were left-handed. Three of these children were included in the fMRI analyses presented here (see later exclusions): 2 in the low ability group, and 1 in the high ability group.

### Design

The non-symbolic numerosity paradigm involved tasks adapted from [11], which assessed accuracy and speed of estimating quantity in dot arrays.

Participants were first trained on the tasks using a laptop computer (see Appendix S1.3 for details of the instructions given to participants). This training took place in a quiet room and lasted approximately 30 minutes. To minimize any potential anxiety and maximize compliance, participants were also familiarised with the scanning environment and procedures in a ‘Mock Scanner’ before going to the main scanning room.

Stimuli in the task were presented in a blocked fashion, with blocks of the experimental task mixed with blocks of the control condition. The complete run included all the stimuli without repetition of items. Each run lasted 4 minutes, excluding instructions which took an additional 42 seconds. We repeated this run three times, randomizing the order of blocks within the runs.

### Stimuli and Procedure

**Figure 2:**
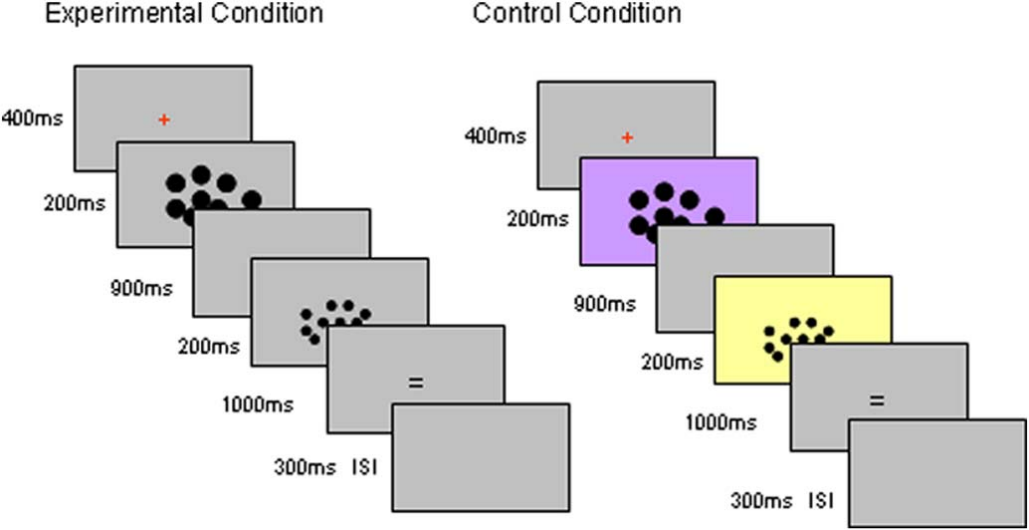
Design. In the experimental condition, participants compared two sets of dots. At first they saw a fixation cross (400 ms). A set of dots were then presented (200 ms), followed by a blank screen (900 ms) and the second set of dots (200 ms). When the second set of dots disappeared, participants pressed the left-hand button if there were more dots in the second sequence than in the first sequence, and a right-hand button if there were fewer dots in the second set. The response window had duration of 1000 ms regardless of the participant's reaction time. An inter-stimulus interval (ISI) of 300 ms interleaved each trial. Total trial length was 3000 ms. In the control task participants were instructed to concentrate on the color of the screen and ignore the dots. The event lengths matched the experimental conditions, but instead of the dot numerosity, the participants now had to compare the background color in the two presentations. The two background colors (turquoise and yellow) were matched on luminosity. When the second colored screen disappeared, participants pressed the left-hand button if the two screens were the same color; and the right-hand button if the two screens were different color.

During the rest period the screen was completely blank. In both the experimental condition and the control condition (see Figure 2) small black dots on a mid-gray background (turquoise or yellow background in the control condition) were presented inside an imaginary square (i.e., the whole screen was mid gray (turquoise or yellow in the control condition), but the dots were confined to a fixed area in the middle of the screen). This gave an appearance of a concentration of the dot array in the centre of the screen. The distribution of the dots was pseudorandom, though they did not touch or overlap. All of the dots in a particular array were of the same size, but the individual dot diameter varied from array to array with three different dot sizes (small, medium, and large) used. The size of the dots was not an experimental variable, but random variation in this aspect of the dot array meant total area covered by the dots was not a reliable cue to numerosity.

In the experimental condition, five comparison ratios were used, each presented in three absolute set sizes. The ratios of the numbers of dots in two consecutive displays were: 1∶2, 2∶3, 3∶4, 4∶5, and 7∶8. The set-sizes in the displays (the numbers of dots in a display) were: small, medium, and large. The stimuli were manipulated (5 ratios×3 set sizes×3 dot sizes) to produce 180 possible trials, each three seconds long (where the specific combination of ratio/set size/dot size were never repeated between the first and the second presentation). The 180 trials were split into 12 trials of each ratio/set-size combination, which were then further split into three 4-trial mini blocks (randomly selected), giving a total of 45 mini blocks. This design was chosen to allow haemodynamic response to plateau between mini-blocks, while avoiding repetition of several trials of the same ratio/set-size combination. The data were analyzed for main effects of ratio (5 levels: 9 mini-blocks each) and set-size (3 levels: 15 mini-block each).

Two blocks of the control task were used: the first block was the length of 9 mini-blocks: 108 sec. (the same length as the ratio experimental condition). The second block of the control task was the length of 6 mini-blocks: 72 sec. (which can be combined with the first control block to produce a control for the set-size experimental condition that consists of 15 mini-blocks).

### Behavioral data analyses

Prior to the imaging data analyses, we analyzed the behavioral data (i.e. the subjects' responses) collected during the scanning session, averaging the data from the three runs. The results of these analyses were used (a) to select the final groups for the fMRI analyses (equated on task performance), and (b) to check whether the sample used in this study shows typical behavioral patterns in terms of accuracy and reaction times.

The consideration of equality in behavioral performance is important because potential differences in activation might not be related to the processes specific to quantity processing aspects of the tasks, but rather could reflect differences in attention or other general processes. Although these differences might be interesting in themselves, the important question in the current study is whether low and high ability groups show different patterns of activation in mathematically relevant areas of the brain while performing the task with comparable accuracy and speed.

### Image Acquisition

The MRI images were collected on a 1.5T GE Excite II system (General Electric, Waukesha, Wisconsin, USA) equipped with TwinSpeed gradients and running 11.0 software. The body coil was used for RF transmission, and the manufacturer's 8-channel head coil for signal reception for all images. After standard localizer and calibration scans, the following sequences were performed: Structural imaging consisted of a T_1_-weighted Inversion Recovery prepared Spoiled Gradient Echo (IR-SPGR) scan, giving whole brain coverage with isotropic 1.1×1.1×1.1 mm voxel in approximately 6 minutes, plus T_2_ weighted and FLAIR (Fluid Attenuated Inversion Recovery) datasets taking a further 4 minutes. The IR-SPGR was acquired for analysis of grey and white matter volumes, while the latter two sets of images were used radiologically to screen for unexpected brain abnormalities. Functional MR images sensitive to blood oxygen level dependent (BOLD) contrast were obtained with a T_2_* -weighted gradient echo-planar imaging sequence with a TR (repetition time) = 3 s, TE (echo time) = 40 ms, excitation flip angle = 90°, FOV (field of view) 24×24 cm, matrix size = 64×64, giving an in-plane pixel size of 3.75×3.75 mm. Forty three 3.3 mm thick axial cuts, parallel to the AC-PC line, covering the whole brain, were collected.

All techniques were chosen for their whole-brain coverage capabilities. The fMRI data were analyzed with software developed at the Institute of Psychiatry (XBAM [21]–[22]), using a nonparametric approach to minimize assumptions (http://brainmap.it; See Appendix S1.4 for details).

### fMRI data analysis

The data analyses were done in four stages: (a) Average (across the three runs) fMRI parameters for each condition were obtained for each individual; (b) The parameters were averaged for all individuals to obtain group activation maps associated with each condition; (c) For each subject, at each voxel, a regression slope was calculated between difficulty level and the corresponding fMRI responses; (d) We then entered these regression slopes to a subsequent analysis of variance to test the existence of a significant main effect of group (high vs. low mathematical ability) on the regression slopes. The technical details for each of the four stages of the analysis are in Appendix S1.4).

## Supporting Information

Appendix S1 (0.05 MB DOC)
