# Supplementary material for: Brain Correlates of Non-Symbolic Numerosity Estimation in Low and High Mathematical Ability Children
Source: PLoS One. 2009 Feb 24;4(2):e4587. doi: 10.1371/journal.pone.0004587 (PMC2642627; doi:10.1371/journal.pone.0004587)
Supplement: Appendix S1 — (0.05 MB DOC) [file pone.0004587.s001.doc]

**Appendix S1**

**S1.1 Measures for selection of participants.**

*Mathematical ability at 7*

When the children were 7*,* teachers assessed their academic achievement in three areas of mathematics at Key Stage 1 of the U.K. National Curriculum (NC), designed for children aged 5 to 7 years [1-2]. The QCA (Qualifications and Curriculum Authority) provides teachers with guidelines for assessments that aim to cover diverse aspects of the three domains: *Using and Applying mathematics*, *Numbers*, and *Shapes, Space and Measures*. A composite of the three aspects of mathematics was created for the selection of participants for this study.

*Mathematical ability at 10*

When the children were 10, they completed a web-based battery of tests that assessed thee aspects of mathematics performance: Understanding Number, Computation and Knowledge, and Non-Numerical processes. The items were based on the National Foundation for Educational Research 5-14 Mathematics Series, which is linked closely to curriculum requirements in the U.K. and the English Numeracy Strategy [3-5]. A composite score was created using the mean of the percentage correct scores of the three tests (77 items in total).

*IQ score at 7*

Two verbal and two non-verbal cognitive measures designed to yield an index of ‘g’ were administered over the telephone. The verbal measures were the Vocabulary (e.g., ‘What does ‘strenuous’ mean?’) and Similarities (e.g., ‘In what way are milk and water alike?’) subtests of the Wechsler Intelligence Scale for Children (WISC-III-UK; [6]). The non-verbal measures were Picture Completion subtest from the Wechsler Scale, in which a child needs to find a missing part in a picture in 20 seconds, and Conceptual Grouping from the McCarthy Scales of Children’s Abilities (MCSA; [7]), which assesses the child’s ability to deal logically with objects, to classify, and to generalize. A composite measure (a mean standardized score) was constructed for the selection for this study.

*IQ score at 10*

Participants at age 10 were tested on a web-based adaptation of two verbal tests: WISC-III Multiple Choice Information (General Knowledge) and WISC-III Vocabulary Multiple Choice [6]. Two non-verbal reasoning tests were also administered as part of the web battery: WISC-III-UK Picture Completion [6] and Raven’s Standard Progressive Matrices [8]. A composite measure (a mean standardized score) was constructed for the selection for this study.

### *Hyperactivity score at 7*

The Strengths and Difficulties Questionnaire (SDQ; [9]) was used to chart hyperactivity in TEDS’ children. We used hyperactivity scores provided by teachers when the children were 7 years of age. If a child scored 6 or above (borderline abnormal to abnormal) on the hyperactivity scale, they were excluded from the pool of possible participants.

**S1.2 Recruitment of participants.**

Participants were part of the Twins’ Early Development Study’s (TEDS) sample. Data from the first cohort of the TEDS’ sample (children born between January 1994 and August 1995) were used for selection for this study. The number of children with available data at 7 or 10 years of age = 14,828. The following exclusion criteria were used: specific medical syndromes such as Down’s syndrome and other chromosomal anomalies, cystic fibrosis, cerebral palsy, hearing loss, autism spectrum disorder, organic brain damage, extreme outliers for birth weight, gestational age**,** maternal alcohol consumption during pregnancy, and special care after birth.

As the TEDS’ sample is population based, participants for the low and high mathematical ability groups were selected based on the quantitative cut-off definition. For the low ability group, the initial pool of participants was composed of all individuals who scored 1.5 SD or more below the population mean on a composite mathematics score at 7 years of age (teacher ratings), and all individuals who scored more than 1.5 SD below the population mean on a composite mathematics score at 10 years of age (web-based individual assessment). High mathematical ability was defined as at least 1SD above the population mean on the same two measures. From this pool, children with low IQ (more than 1SD below the population mean) and high Hyperactivity scores (above 6) were excluded (see Appendix S1.1 for the description of all measures).

From the remaining large pool of children 39 low mathematical ability and 29 high mathematical ability children were selected semi-randomly. Only one twin was selected from each family. In cases where both twins met the selection criteria, the twin with the highest or lowest scores was selected (for the high and low ability groups respectively). The priority was given to those children who met the criteria for selection at 7 and 10 years of age.

Sixty-eight information packs were mailed out to the families. The information packs contained: (1) a short letter explaining the main objectives of the study; (2) information sheet for parents with detailed description of the study; (3) child information sheet; (4) questions and answers sheet about Magnetic Resonance Imaging; (5) parent consent form; (6) child consent form; (7) MRI safety questionnaire; (8) child fun activity sheet. The families were asked to read the information pack carefully, and then fill in the parent and child consent forms and send them back in the provided envelope. If the family agreed to participate, they were also asked to return the completed MRI safety questionnaire which screens for possible unsuitability for the MRI procedure.

Thirty-two responded positively by telephone and by returning parent and child consent forms. 11 families did not respond in time for selection. 25 families declined to participate on this occasion or were unsuitable for MRI. Researchers then contacted all families who responded positively by telephone in order to book appointments and respond to queries if necessary.

Because the participants in this study were schoolchildren, the slots allocated for the study were four Saturdays and eight weekdays during two half terms in the period between 5 February and 18 June, 2005. 28 children (14 low and 14 high mathematical ability children) were able to attend the scanning session during this time period.

Eleven of the families lived within London, which meant that most families had to travel some distance (>25 miles) to attend. There was no known drop-out due to traveling constraints.

**S1.3 Instructions and practice for computer tasks.**

The children were told that they would do 2 types of tasks: DOT TASK and COLOR TASK. They were also instructed that there would be a gap between the tasks during which they should wait for the next task while trying not to move. The children were told that when all the tasks are finished, they would see ‘END’. Participants were asked to use fingers on both hands. They were also asked not to worry if they were not sure, but just try their best. They were then given specific instructions followed by practice for each of the tasks as follows.

“Sometimes the computer will show you “DOT TASK” in the middle of the screen. When you see this, you will have to compare two sets of dots. At first you will see a + on the computer screen. It will help you to focus. It will look like this: (EXAMPLE). You will see one set of dots and then another set of dots. The second set will appear right after the first one. The dots will flash very quickly. They will look like this: (EXAMPLE). When the second set of dots disappears, press the left-hand button if the second set had **more** dots than the first one. Press the right-hand button if the second set had **fewer** dots than the first one. Press as fast as you can.”

“Sometimes the computer will show you “COLOUR TASK” in the middle of the screen. When you see this, you will have to look at the color of the screen. At first you will see a + on the computer screen. It will help you to focus. It will look like this: (EXAMPLE). You will see two colored screens with some dots on them one after another. The colors will flash very quickly. They will look like this: (EXAMPLE). When the second colored screen disappears, press the left-hand button if the two screens were the same color. Press the right-hand button if the two screens were different color. Press as fast as you can.”

Feedback was given during the initial practice phase and instructions were repeated when necessary. Then the children practiced the tasks one after another (in a random order) as they would be presented in the scanner. This session was interrupted if it became clear that the child did not understand the tasks, in which case the instructions were repeated, and the practice resumed. Overall it took between 15 and 45 minutes to train children on the tasks.

The children were then instructed that during the scanning session they would sometimes be asked not to do anything for several minutes, but to relax and try not to move. This was necessary for the acquisition of the structural images which were interspersed between the functional imaging blocks.

**S1.4** **XBAM analyses.**

fMRI analysis, in its most common forms, requires fitting of a time series model at many thousands of intracerebral voxels for each individual. Most assessments of the significance of the resulting model fits are based on normal theory and the validity of the normality assumption is not often tested. The XBAM software makes no such assumptions; instead, it uses median statistics to control outlier effects and employs permutation rather than normal theory-based inference. Furthermore, its most common test statistic is computed by standardising for individual difference in residual noise before embarking on second level, multi-subject testing using robust permutation-based methods. This allows a mixed effects approach to analysis. A recent paper [10] has conducted a detailed analysis of the validity and impact of normal theory-based inference in fMRI in a large number of subjects (81). They have found substantial deviations from normality in a significant number (22%) of intracerebral voxels using the most common measure of response size (unstandardized beta) used in fMRI analysis. We have recently confirmed that a similar level of non-normality occurs in our own datasets (information available from MB). Thirion et al. [10] recommend a mixed effects rather than simple random effects analysis (i.e. an approach in which differences in intra-subject residual error as well as inter-subject variability are specifically included in the analysis). To deal with the issue of non-normality and its (unknown) effects on parametric hypothesis testing, they recommend permutation-based inference and cluster or parcel level rather than voxel-level inference. Haysaka and Nichols [11], in comparing Gaussian random field based and permutation based cluster analysis have also strongly recommended permutation-based analysis for greater robustness and minimal assumptions as they too were concerned about (usually untested) assumptions of data normality.

Data were first corrected for subject motion [12] and then smoothed using a Gaussian filter (FWHM 7.2 mm) chosen to improve signal-to-noise ratio over the spatial neighborhood of each voxel. Responses to the experimental paradigms were then detected by time-series analysis using a linear model in which each component of the experimental design was convolved separately with two gamma variate functions (peak responses at 4 and 8 sec, respectively) to permit variability in the haemodynamic delay. The method of Friman, Borga, Lundberg, & Knutsson [13] was used to constrain model fits to those deemed physiologically plausible. Following computation of the model fit, a goodness of fit statistic was computed. This consisted of the ratio of the sum of squares of deviations from the mean image intensity due to the model (over the whole time series) to the sum of squares of deviations due to the residuals (SSQratio). This addresses the problem inherent in the use of the F statistic that the residual degrees of freedom are often unknown in fMRI time series due to the presence of colored noise in the signal. It has also been shown by Edgington ([14], page 61) to behave equivalently to F under permutation testing. Following computation of the observed SSQratio at each voxel, the data were permuted by the wavelet-based method described and extensively characterized in Bullmore et al. [15] which permits the data-driven calculation of the null distribution of SSQratios under the assumption of no experimentally-determined response. This distribution can then be used to create a threshold for activation maps at any desired type I error rate. In addition to the SSQratio, the percentage BOLD change was also calculated from the model fit at each voxel.

The observed and randomized SSQratio data for each individual was transformed into standard space of Talairach and Tournoux [16] and group maps of activated regions were computed using the median observed and randomized SSQratio data as described by Brammer et al. [17]. Such normalization should be valid even in children, due to very small differences (relative to the resolution of fMRI data) in the spatial correspondence among several brain loci between young children and adults after a standard, nonlinear transformation that warped child and adult fMRI data into a common adult Talairach space [18]. Permutation methods and median statistics were employed to allow exact computation of p values with minimal assumptions and minimization of outlier effects. The hierarchical method of analysis used above also allows separate treatment of intra- and inter-individual variance. After extension of inference from voxel to cluster level using the method described in detail by Bullmore, Suckling et al. [19], thresholds for the resulting cluster maps were selected to give < 1 expected type I error cluster per whole brain volume to make interpretation of maps as intuitive as possible.

For each subject, at each voxel, a regression slope was calculated between ratio difficulty level and the corresponding fMRI responses. These regression slopes were then entered into a subsequent analysis of variance to test the existence of a significant main effect of group (high vs. low mathematical ability) on the regression slopes.

**References**

1. Qualifications and Curriculum Authority (1999) QCA Key Stage 1: Assessment and reporting arrangements. Great Britain: Qualifications and Curriculum Authority.

2. Qualifications and Curriculum Authority (2003) QCA Key Stage 1: Assessment and reporting arrangements. Great Britain: Qualifications and Curriculum Authority.

3. nferNelson (1994) *Maths 5-14 Series*. London: nferNelson Publishing Company Ltd.

4. nferNelson (1999) *Maths 5-14 Series*. London: nferNelson Publishing Company Ltd.

5. nferNelson (2001) *Maths 5-14 Series*. London: nferNelson Publishing Company Ltd.

6. Wechsler D (1992) Wechsler intelligence scale for children - Third Edition UK (WISC-IIIUK) Manual. London The Psychological Corporation.

7. McCarthy D (1972) McCarthy Scales of Children’s Abilities. New York: the Psychological Corporation.

8. Raven J C, Court JH, & Raven J (1996) Manual for Raven's Progressive Matrices and Vocabulary Scales. Oxford: Oxford University Press.

9. Goodman R (1997) The Strengths and Difficulties Questionnaire: A research note. Journal of Child Psychology & Psychiatry & Allied Disciplines 38(5): 581-586.

10. Thirion B, Pinel P, Meriaux S, Roche A, Dehaene S, et al. (2007) Analysis of a large fMRI cohort: Statistical and methodological issues for group analysis. Neuroimage 35: 105-120.

11. Hayasaka S, & Nichols TE (2003) Validating cluster size inference: random field and permutation methods. Neuroimage 20: 2343-2356.

12. Bullmore ET, Brammer MJ, Rabe-Hesketh S, Curtis V, Morris RG, et al. (1999) Methods for diagnosis and treatment of stimulus-correlated motion in generic brain activation studies using fMRI. Human Brain Mapping 7: 38-48.

13. Friman O, Borga P, Lundberg P, & Knutsson H (2003) Adaptive analysis of fMRI data. Neuroimage 19: 837-845.

14. Edgington ES (1995) Randomization Tests. Marcel Dekker Inc., New York Basel Hong Kong.

15. Bullmore ET, Long C, Suckling J, Fadili J, Calvert GA, et al. (2001) Coloured noise and computational inference in neurophysiological (fMRI) time series analysis: Resampling methods in time and wavelet domains. Human Brain Mapping 12: 61-78.

16. Talairach J, & Tournoux P (1988) Co-planar stereotaxic atlas of the human brain. Thieme New York.

17. Brammer M J, Bullmore ET, Simmons A, Williams SCR, Grasby PM, et al. (1997) Generic brain activation mapping in fMRI: a nonparametric approach. Magnetic Resonance Imaging 15: 763-770.

18. Cantlon JF, Brannon EM, Cater EJ, & Pelphrey KA (2006) Functional imaging of numerical processing in adults and 4-y-old children. PLoS Biology 4(5): 0844-0854.

19. Bullmore ET, Suckling J, Overmeyer S, Rabe-Hesketh S, Taylor E, et al. (1999) Global, voxel and cluster tests, by theory and permutation, for a difference between two groups of structural MR images of the brain. IEEE Trans. Med. Imaging 18: 32-42.
